# Sistemas de vigilancia de anomalías congénitas en América Latina y el Caribe: presente y futuro

**DOI:** 10.26633/RPSP.2019.44

**Published:** 2019-05-24

**Authors:** Pablo Durán, Rosa Liascovich, Pablo Barbero, María Paz Bidondo, Boris Groisman, Suzanne Serruya, Luis Andrés de Francisco, Francisco Becerra-Posada, Amparo Gordillo-Tobar

**Affiliations:** 1 Centro Latinoamericano de Perinatología Centro Latinoamericano de Perinatología Salud de la Mujer y Reproductiva, Organización Panamericana de la Salud/Organización Mundial de la Salud Montevideo Uruguay Centro Latinoamericano de Perinatología, Salud de la Mujer y Reproductiva, Organización Panamericana de la Salud/Organización Mundial de la Salud, Montevideo, Uruguay.; 2 Red Nacional de Anomalías Congénitas de Argentina (RENAC) Red Nacional de Anomalías Congénitas de Argentina (RENAC) Centro Nacional de Genética Médica, ANLIS Carlos Malbrán, Ministerio de Salud y Desarrollo Social Buenos Aires Argentina Red Nacional de Anomalías Congénitas de Argentina (RENAC), Centro Nacional de Genética Médica, ANLIS Carlos Malbrán, Ministerio de Salud y Desarrollo Social, Buenos Aires, Argentina.; 3 Organización Panamericana de la Salud/Organización Mundial de la Salud Organización Panamericana de la Salud/Organización Mundial de la Salud WashingtonDC Estados Unidos de América Organización Panamericana de la Salud/Organización Mundial de la Salud, Washington, DC, Estados Unidos de América.; 4 Banco Mundial Banco Mundial WashingtonD.C. Estados Unidos de América Banco Mundial, Washington, DC, Estados Unidos de América.

**Keywords:** Anomalías congénitas, servicios de vigilancia epidemiológica, América Latina, Región del Caribe, Congenital abnormalities, epidemiologic surveillance services, Latin America, Caribbean Region, Anormalidades congênitas, serviços de vigilância epidemiológica, América Latina, Região do Caribe

## Abstract

**Objetivos.:**

Conocer la disponibilidad de los sistemas nacionales de vigilancia de anomalías congénitas en América Latina y el Caribe y describir sus características.

**Métodos.:**

Estudio transversal mediante una encuesta semiestructurada y autoadministrada en línea remitida en el 2017 por las representaciones locales de la Organización Panamericana de la Salud a las autoridades de los ministerios de salud de todos los países de América Latina y el Caribe. La encuesta recabó información sobre la disponibilidad de un sistema nacional de vigilancia de anomalías congénitas en el país y sus características.

**Resultados.:**

Once países cuentan con sistema nacional de vigilancia de anomalías congénitas: Argentina, Colombia, Costa Rica, Cuba, Guatemala, México, Panamá, Paraguay, República Dominicana, Uruguay y Venezuela. Los sistemas tienen características heterogéneas: 6 son sistemas de base hospitalaria; 10 incluyen en su definición de caso los nacidos vivos y los fetos muertos. En todos los sistemas de vigilancia se incluyen los casos con anomalías mayores y menores, excepto en Argentina, Colombia y Guatemala que solo registran anomalías congénitas mayores. Solo Argentina, Costa Rica y Uruguay elaboran informes periódicos que consolidan y presentan los resultados de la vigilancia; los registros de Argentina y Costa Rica disponen de manuales operativos.

**Conclusiones.:**

Se comprobó la aún escasa disponibilidad de sistemas nacionales de vigilancia de anomalías congénitas en América Latina y el Caribe y su elevada heterogeneidad. Es prioritario avanzar hacia la expansión y el fortalecimiento de este tipo de vigilancia en nuestros países.

## SUMMARY

**Objectives.:**

To determine the availability of national systems for surveillance of birth defects in Latin America and the Caribbean and describe their characteristics.

**Methods.:**

Cross-sectional study based on a semi-structured, self-administered online survey sent in 2017 by local representative offices of the Pan American Health Organization to authorities at the ministries of health of all countries in Latin America and the Caribbean. The survey obtained information on the availability and characteristics of national systems for surveillance of birth defects in each country.

**Results.:**

Eleven countries have a national system for surveillance of birth defects: Argentina, Colombia, Costa Rica, Cuba, Dominican Republic, Guatemala, Mexico, Panama, Paraguay, Uruguay, and Venezuela. These systems have heterogeneous features: six are hospital-based; 10 include both live births and stillbirths in their case definition. All the surveillance systems include cases with severe and minor defects, except in Argentina, Colombia, and Guatemala, where only severe birth defects are recorded. Only Argentina, Costa Rica, and Uruguay prepare periodic reports that consolidate and present the results of surveillance. The registries in Argentina and Costa Rica have operational manuals.

**Conclusions.:**

The availability of national systems for surveillance of birth defects remains limited and highly heterogeneous in Latin America and the Caribbean. Priority should be given to continued expansion and strengthening of this type of surveillance in these countries.

## RESUMO

**Objetivos.:**

Conhecer a existência de sistemas nacionais de vigilância de anomalias congênitas na América Latina e Caribe e descrever as características destes sistemas.

**Métodos.:**

Estudo transversal realizado por meio de uma pesquisa online com uso de questionário semiestruturado e autoaplicado enviado no 2017 pelas representações locais da Organização Pan-Americana da Saúde (OPAS) às autoridades dos ministérios da Saúde em todos os países da América Latina e Caribe. Foram coletados dados sobre a existência de sistema nacional de vigilância de anomalias congênitas e as características destes sistemas.

**Resultados.:**

Onze países possuem um sistema nacional de vigilância de anomalias congênitas: Argentina, Colômbia, Costa Rica, Cuba, Guatemala, México, Panamá, Paraguai, República Dominicana, Uruguai e Venezuela. Os sistemas são de natureza heterogênea, seis são baseados em registros hospitalares e 10 incluem nascidos vivos e morte fetal na definição de caso. A maioria dos sistemas de vigilância abrange casos de anomalias congênitas maiores e menores, exceto na Argentina, Colômbia e Guatemala em que são registradas apenas as anomalias congênitas maiores. A Argentina, Costa Rica e Uruguai são os únicos países que apresentam relatórios periódicos reunindo os achados da vigilância e a Argentina e Costa Rica possuem manuais operacionais para o registro.

**Conclusões.:**

Verificou-se que, na América Latina e no Caribe, os sistemas nacionais de vigilância de anomalias congênitas são ainda em um pequeno número e bastante heterogêneos. É uma prioridade expandir e reforçar esta forma de vigilância nos países da região.

En el mundo, la mortalidad infantil muestra una concentración relativa creciente en el período neonatal a expensas de reducciones en las defunciones a edades mayores (1-3). La mortalidad neonatal en el mundo descendió 37% entre 1990 y 2012: de 33 a 21 defunciones por 1 000 nacidos vivos, respectivamente; los mayores descensos se registraron en las defunciones en el período posneonatal, superiores a 50%, aunque con importantes variaciones entre regiones (4). En la Región de las Américas, el descenso en la tasa de mortalidad neonatal fue de 57,9% entre 1990 y 2014 (de 22,1 a 9,3 defunciones neonatales por 1 000 nacidos vivos, respectivamente) (5).

En cuanto a la contribución relativa de las causas específicas de mortalidad neonatal, también se observan variaciones entre poblaciones. A los defectos congénitos les corresponde una parte considerable de la carga de enfermedad y acumulan en conjunto entre 25,3 y 38,8 millones de años de vida perdidos ajustados por discapacidad en el mundo (6). En la Región de las Américas, los defectos congénitos se encuentran entre las principales causas de muerte y, si bien en los países con menores ingresos los defectos congénitos representan en términos relativos menos de 5% de las causas de mortalidad infantil, en los países de mayores ingresos a este grupo de causas se asocia el 30% de las muertes registradas antes del año de vida (6). Además, los defectos congénitos constituyen una de las principales causas de discapacidad en la niñez. La prevalencia de discapacidad moderada y grave en menores de 14 años en países con bajos y medianos ingresos de la Región se ha estimado en 4,5% y una proporción considerable de ella se atribuye a defectos congénitos (7).

En el año 2010, la Asamblea Mundial de la Salud adoptó una resolución especial sobre los defectos congénitos (8) en la que se insta a los Estados Miembros a:

fomentar la sensibilización acerca de la importancia de los defectos congénitos como causa de morbilidad y mortalidad infantiles; establecer prioridades, consignar recursos y formular planes y actividades para integrar intervenciones eficaces de prevención de los defectos congénitos y su atención

desarrollar sistemas de vigilancia orientados a contar con datos sobre los defectos congénitos en el marco de los sistemas nacionales de información sanitaria

crear capacidades en materia de prevención y tratamiento de los defectos congénitos y de atención a los niños, así como prestar apoyo a las familias con niños con defectos congénitos y discapacidades asociadas, y velar por que los niños con discapacidad reciban la rehabilitación y el apoyo apropiados

intensificar las investigaciones y los estudios sobre la etiología, el diagnóstico y la prevención de los principales defectos congénitos y promover la cooperación internacional para combatir estas afecciones.

La vigilancia, el monitoreo y la evaluación de trastornos que, como los defectos congénitos, contribuyen sustancialmente a la carga de mortalidad, morbilidad y discapacidad (8-11) son estrategias prioritarias que permiten valorar tendencias, diseñar intervenciones y dar respuesta a situaciones emergentes. Un claro ejemplo de ello ha sido la emergencia de la epidemia por el virus del Zika, que ocasionó más de 700 000 casos de infección (12) y más de 3 700 casos registrados oficialmente de síndrome congénito asociado al Zika en la Región de las Américas (13).

En algunos países de la Región existen registros de defectos congénitos que, aun cuando su propósito no es alcanzar representatividad nacional total, han contribuido a la implementación de actividades de vigilancia. También funcionan registros y organizaciones internacionales y regionales, como International Clearinghouse for Birth Defects Surveillance and Research (ICBDSR), un consorcio de programas de vigilancia de diferentes áreas geográficas (14) —con instituciones afiliadas en Argentina, Chile (región de Maule), Colombia (Bogotá y Cali), Cuba y México—, y el Estudio Colaborativo Latinoamericano de Malformaciones Congénitas (ECLAMC), una red sudamericana que inició sus actividades de vigilancia de defectos congénitos a finales de la década de 1960 (15). No obstante, no existe información detallada acerca de sistemas de vigilancia de defectos congénitos de alcance nacional que contribuyan a la formulación de políticas y la vigilancia sanitaria en los países de la Región.

Debido a estas carencias, el objetivo del presente estudio fue conocer la disponibilidad de los sistemas nacionales de vigilancia de anomalías congénitas en América Latina y el Caribe y describir sus características.

## MATERIALES Y MÉTODOS

Se realizó un estudio transversal a partir de una encuesta remitida por las representaciones locales de la Organización Panamericana de la Salud (OPS) a las autoridades de los ministerios de salud de todos los países de América Latina y el Caribe. Desde el Centro Latinoamericano de Perinatología, Salud de la Mujer y Reproductiva (CLAP) se remitió la información acerca de las características y el alcance del estudio a los asesores de la OPS en cada uno de los Estados Miembros, responsables del área de salud perinatal, junto con el vínculo para acceder a la encuesta en línea. Los asesores, a su vez, remitieron esa información a los responsables de salud perinatal de los ministerios de salud para que la completaran si el país contaba con un sistema nacional de vigilancia de defectos congénitos.

Se consideró que se disponía de tal sistema cuando en el país existía un mecanismo de coordinación y consolidación, por parte de las autoridades nacionales, de los resultados de la vigilancia a nivel institucional, aun cuando la cobertura no se extendiera a la totalidad de los nacimientos del país. A los fines de este estudio, la vigilancia de defectos congénitos a nivel institucional y la remisión de datos a registros regionales o internacionales no se consideraron como sistemas nacionales de vigilancia.

La encuesta semiestructurada —disponible y autoadministrada en línea a través de la plataforma Survey Monkeyhttp://www.surveymonkey.com — se aplicó en inglés o español, según el idioma del país. Luego del envío inicial de la encuesta, se reiteró el pedido en dos oportunidades a las autoridades de los países que no habían respondido; el período de recolección de datos fue de julio a noviembre de 2017. La encuesta empleada está a disposición de los interesados previa solicitud al autor de correspondencia de este artículo.

En la encuesta se recabó información sobre la disponibilidad de un sistema nacional de vigilancia de anomalías congénitas en el país y se indagó sobre la dependencia institucional del sistema, la información de contacto y las características del diseño metodológico (cuadro 1). En caso de no contar con ese sistema, no se completaba la encuesta.

Las dimensiones valoradas en la encuesta fueron: el tipo de vigilancia, entendido como la base de referencia del sistema de vigilancia (poblacional u hospitalario); la cobertura del sistema de vigilancia (nacional con datos de todas las provincias o departamentos del país, o provincial cuando solo abarcaba una provincia o departamento); la dependencia administrativa de los establecimientos que reportan (públicos, de la seguridad social o privados); el método de recolección (vigilancia activa cuando los miembros de la coordinación central del sistema concurren a los establecimientos y revisan las historias clínicas de los casos, vigilancia pasiva cuando los equipos de salud de los establecimientos son los que recolectan los datos y los envían a la coordinación, o vigilancia híbrida cuando cuenta con “referentes” o líderes en los establecimientos de salud que coordinan la recolección de datos). Estas variables se definieron según el manual para gestores de programas para la vigilancia de anomalías congénitas (16). Se indagó también sobre el sistema de codificación utilizado, el número aproximado de nacimientos anuales incluidos en la vigilancia referida, los desenlaces del embarazo considerados para la vigilancia (nacidos vivos, fetos muertos o terminaciones electivas del embarazo por anomalías fetales), la edad límite para reportar una anomalía congénita (e.g. hasta el alta de la maternidad, 1 año de vida o 6 años de vida), si cuentan con manual operativo u otros materiales de apoyo para la recolección de datos, y si se realizan informes periódicos para difundir la información.

Una vez completado el período establecido para el llenado de la encuesta en línea, uno de los investigadores corroboró la no disponibilidad de sistema de vigilancia en los países que no completaron la encuesta en línea. Se comprobó la consistencia de los datos recolectados y se realizó el análisis descriptivo de los mismos de acuerdo con las categorías y escalas de medición definidas para cada variable.

**CUADRO 1:** Variables utilizadas en el relevamiento de sistemas de vigilancia de anomalías congénitas, América Latina y el Caribe, 2017

Variable	Categorías
Tipo de vigilancia	Base hospitalaria: la información se recolecta en maternidades seleccionadas y la cobertura corresponde a los nacimientos que ocurren en esas maternidades Base poblacional: la cobertura abarca todos los nacimientos de mujeres residentes en un área determinada, con independencia del lugar donde ocurre el nacimiento
Fuente de datos	Maternidades Maternidades y otras instituciones
Cobertura	Nacional o provincial
Dependencia de los establecimientos que reportan	Establecimientos públicos, de la seguridad social o privados
Método de recolección	Pasivo: la información se reporta desde las instituciones participantes, sin revisión por una coordinación central Activo: personal de la coordinación central visita las instituciones participantes y recoge información sobre los casos afectados Híbrido: la información se reporta desde las instituciones participantes a la coordinación central, donde se realiza la revisión de los casos
Sistema de codificación	CIE-10 CIE-10 con la modificación del RCPCH/BPA^a^ Sistema propio Otro (especifique)
Número de nacidos vivos por año	Número de nacidos vivos por año en el sistema Número de nacidos vivos por año en el país
Desenlaces del embarazo	Nacidos vivos Fetos muertos Terminaciones electivas del embarazo por anomalías fetales
Definición de casos que ingresan al sistema	Anomalías mayores o menores Solo anomalías mayores Otro (especifique)
Edad límite para reportar una anomalía congénita	Alta de la maternidad 1 semana de vida 1 mes de vida 6 meses de vida 1 año de vida 6 años de vida
Manual operativo	Tiene No tiene
Tipo de formulario del sistema	En papel Formato electrónico
Modo de envío de los datos	Por correo postal Por correo electrónico A través de una página web Otro (especifique)
Adónde se envían los datos	A una coordinación central nacional A nodos intermedios
¿Existe un equipo de coordinación a nivel central?	Sí No
Fuente del denominador	Nacimientos en los hospitales participantes Estadísticas vitales Otro (especifique)
¿Se elaboran informes periódicos?	Sí No
¿Cuentan con un atlas fotográfico?	Sí No

***Fuente:*** elaboración propia.

a RCPCH/BPA: Royal College of Paediatrics and Child Health/British Paediatric Association (18).

## RESULTADOS

Según los resultados de la encuesta, 11 países disponían de sistemas nacionales de vigilancia de anomalías congénitas: Argentina, Colombia, Costa Rica, Cuba, Guatemala, México, Panamá, Paraguay, República Dominicana, Uruguay y Venezuela. En el momento de la encuesta, en todos los casos el sistema de vigilancia dependía del ministerio o secretaria de salud, y los datos se centralizaban en una coordinación nacional. En el cuadro 2 se resumen las características de los sistemas de vigilancia de cada país.

**CUADRO 2:** Características de los sistemas de vigilancia con cobertura nacional^a^, América Latina y el Caribe, 2017

País	Nombre del programa	Año de inicio	Tipo de vigilancia^b^	Dependencia de los establecimientos que reportan	Método de recolección (sistema de codificación)	Nacidos vivos incluidos en el sistema de vigilancia (% de cobertura en relación con el total de nacidos vivos en el país)	Edad límite para reportar una anomalía congénita	Fuente del denominador	Casos que ingresan al sistema
Argentina	Red Nacional de Anomalías Congénitas de Argentina (RENAC)	2010	Hospitalaria	Establecimientos públicos y privados^c^	Híbrido (CIE-10)^d^	300 000 (39%)	Alta de la maternidad	Nacimientos en los hospitales participantes	Solo anomalías mayores
Colombia	Vigilancia de los Defectos Congénitos	2010	Poblacional	NE^e^	Pasivo (CIE-10)	660 999 (100%)	1 año de vida	Estadísticas vitales	Solo anomalías mayores
Costa Rica	Centro de Registro de Enfermedades Congénitas (CREC)	1987	Poblacional	Establecimientos públicos y privados	Híbrido (CIE-10)^d^	70 000 (100%)	1 año de vida	Estadísticas vitales	Anomalías mayores y menores
Cuba	Registro Cubano de Malformaciones Congénitas (RECUMAC)	1985	Hospitalaria	Todas las maternidades del país	Híbrido (CIE-10)^d^	120 000 (100%)	Alta de la maternidad	Estadísticas vitales	Anomalías mayores y menores
Guatemala	Protocolo de Vigilancia de Anomalías Congénitas	2017	Hospitalaria	Establecimientos públicos	Híbrido (CIE-10)	155 000 (40%)	1 mes de vida	Nacimientos en los hospitales participantes	Solo anomalías mayores
México	Sistema de Vigilancia Epidemiológica de Defectos del Tubo Neural y Defectos Craneofaciales	1999	Poblacional	NE	Híbrido (CIE-10)	1 404 (0,07%)	1 año de vida	Estadísticas vitales	Anomalías mayores y menores
Panamá	Sistema de Detección de Malformaciones del Tubo Neural	2013	NE	NE	NE	NE	NE	NE	NE
Paraguay	Registro Nacional de Defectos Congénitos	2016	Hospitalaria	Establecimientos públicos	Pasivo (CIE-10)	80 000 (70")	1 año de vida	Nacimientos en los hospitales participantes	Anomalías mayores y menores
República Dominicana	Sistema Nacional de Vigilancia Epidemiológica	2016	Hospitalaria	NE	Híbrido (CIE-10)	193 000 (100%)	Alta de la maternidad	Estimaciones de la Oficina Nacional de Estadística	Anomalías mayores y menores
Uruguay	Registro Nacional de Defectos Congénitos y Enfermedades Raras (RNDCER)	2011	Poblacional	NE	Híbrido (CIE-10)	28 000 (58%)	6 años de vida	Estadísticas vitales	Anomalías mayores y menores
Venezuela	Registro de Paciente con Enfermedades por Errores Innatos del Metabolismo	2017	Hospitalaria	Establecimientos públicos	Pasivo (CIE-10)	NE	6 años de vida	Estadísticas vitales	Anomalías mayores y menores

***Fuente:*** elaboración propia con datos obtenidos en la encuesta; también de Argentina: http://www.anlis.gov.ar/cenagem/?page_id=584 y de Costa Rica: https://www.inciensa.sa.cr/actualidad/Informes%20de%20vigilancia.aspx#HERMES_TABS_1_5

a En todos los casos los desenlaces incluidos son los nacidos vivos y los fetos muertos, excepto en Cuba, México y Uruguay, que incluyen además las terminaciones electivas del embarazo.

b Hospitalaria: fuente de datos de maternidades solamente; poblacional: fuente de datos de maternidades y otras instituciones. La cobertura es nacional, excepto en Guatemala, Panamá y Venezuela que no lo especificaron explícitamente.

c Incluye los establecimientos de la seguridad social.

d CIE-10 (17) y la modificación del Royal College of Paediatrics and Child Health (18).

e NE: no especificado por el país.

Los sistemas identificados presentaron características diferentes entre sí. Ocho de los sistemas comenzaron a operar en 2010 o después, excepto Cuba, que comenzó en 1985, Costa Rica en 1987 y México en 1999. Cuatro de los sistemas eran de base poblacional; las fuentes de datos reconocidas son las maternidades y otras instituciones de salud (6 países) y de cobertura nacional (8 países).

El método de recolección de casos se describió como híbrido, con excepción de los sistemas de Colombia, Paraguay y Venezuela, donde es pasivo. Excepto Panamá, que no remitió información al respecto, todos los países codifican las anomalías congénitas según la 10.ª revisión de la Clasificación Estadística Internacional de Enfermedades y Problemas Relacionados con la Salud (CIE-10) (17), aunque Argentina, Costa Rica y Cuba usan, además, la adaptación del Royal College of Paediatrics and Child Health (18).

Todos los sistemas incluyen nacidos vivos y fetos muertos, excepto Venezuela que solo considera a los nacidos vivos; en los registros de Cuba, México y Uruguay también se incluyen los casos de terminación electiva del embarazo por anomalías congénitas.

En relación con la edad límite para reportar una anomalía congénita, en tres países (Argentina, Cuba y República Dominicana) se registran las anomalías congénitas hasta el momento del alta de los recién nacidos en las maternidades, en Guatemala hasta el mes de vida, en cuatro países (Colombia, Costa Rica, México y Paraguay) hasta 1 año de edad y en dos países (Uruguay y Venezuela) hasta los 6 años de vida; Panamá no envió información al respecto. En todos los sistemas de vigilancia se incluyen los casos con anomalías mayores y menores, excepto en Argentina, Colombia y Guatemala que solo registran anomalías congénitas mayores.

Solo tres registros elaboran informes periódicos que consolidan los resultados de la vigilancia: Argentina, Costa Rica y Uruguay. Solamente los registros de Argentina y Costa Rica confirmaron la disponibilidad de manuales operativos.

## DISCUSIÓN

Los resultados de esta encuesta muestran que en América Latina y el Caribe, si bien algunos países cuentan con sistemas nacionales de vigilancia de anomalías congénitas consolidados, la mayoría de ellos aún carece de estos o se encuentran en fase de desarrollo. Solo tres países generan informes periódicos (Argentina, Costa Rica y Uruguay). De ellos, Argentina y Costa Rica han realizado publicaciones científicas utilizando los datos de los sistemas de vigilancia (19, 20). Por otra parte, solo tres sistemas (los de Argentina, Cuba y Costa Rica) comparten regularmente sus datos con consorcios internacionales, como el ICBDSR.

En la Región de las Américas se han desarrollado diferentes iniciativas tendientes a implementar registros de vigilancia a lo largo de más de 50 años. Como antecedentes relevantes en el desarrollo de los registros de vigilancia en América Latina cabe destacar el ECLAMC (15), ya mencionado, y el Sistema Informático Perinatal (SIP) (21).

El ECLAMC, iniciado en 1967, es un programa de investigación con diseño de casos y controles que ha generado más de 300 publicaciones científicas. Si bien existen establecimientos en los diferentes países que registran datos en el marco del ECLAMC, estos no alcanzan la representatividad nacional, a menos que el país cuente con un sistema nacional de vigilancia específica que los incluya junto a otros. Por ello, el hecho de que en el marco del presente estudio muchos países hayan informado que no disponen de un sistema nacional de vigilancia no significa que en ellos no se realicen acciones de este tipo. Se debe resaltar la diferencia entre disponer de registros de vigilancia en una o más instituciones y disponer de un sistema nacional de vigilancia de anomalías congénitas, hacia los cuales se enfocó el presente trabajo.

El SIP, creado hace más de 25 años por iniciativa del CLAP, reúne los datos de todas las mujeres embarazadas y sus hijos desde la primera visita prenatal hasta el alta de ambos después del parto. El SIP, una de las herramientas de la OPS para mejorar la calidad de la atención a las madres y los recién nacidos, funciona en la mayoría de los países de América Latina y el Caribe y cuenta con un módulo específico para el registro de anomalías congénitas (16).

Recientemente el CLAP —en colaboración con los Centros para el Control y la Prevención de Enfermedades (CDC), de los Estados Unidos de América, y el ICBDSR— promovieron un conjunto de actividades dirigidas a mejorar la prevención y el tratamiento de las anomalías congénitas mediante el fortalecimiento y la creación de sistemas de vigilancia, principalmente en los países de bajos y medianos ingresos.

Una de sus primeras actividades fue la realización del Taller sobre Vigilancia de Anomalías Congénitas y Partos Prematuros, organizado conjuntamente con otras instituciones nacionales —el Instituto Costarricense de Investigación y Enseñanza en Nutrición y Salud (INCIENSA), de Costa Rica; la Universidad Javeriana, de Colombia; y la Red Nacional de Anomalías Congénitas (RENAC), de Argentina— e internacionales —los ya mencionados ICBDSR, CLAP y CDC—, que incluyó un curso previo con un componente virtual y otro presencial. Este  taller, realizado en 2015 en San José, Costa Rica, y replicado en el 2016 en Bogotá, Colombia, se dirigió a epidemiólogos y decisores de los ministerios de salud, así como a pediatras seleccionados de los 18 países participantes (Argentina, Bolivia, Brasil, Chile, Colombia, Costa Rica, Cuba, Ecuador, El Salvador, Guatemala, Honduras, Nicaragua, Panamá, Paraguay, Perú, República Dominicana, Uruguay y Venezuela). El propósito de estos talleres era brindar herramientas a líderes y profesionales para fortalecer la vigilancia de las anomalías congénitas. En una segunda etapa, que comenzó en 2017 en El Salvador y Panamá, se inició la capacitación de profesionales nacionales con el objetivo de promover su vinculación con la OPS, las autoridades nacionales y los actores clave de sus países a fin de elevar el nivel de sensibilización e involucrar a la red de servicios y profesionales para conformar el sistema de vigilancia nacional. Este proceso se encuentra en etapa de expansión a otros países.

Entre las limitaciones de este trabajo se podría mencionar que solo 11 países respondieron la encuesta; no obstante, se corroboró que los que no respondieron no contaban con sistemas nacionales de vigilancia, de modo que los resultados reflejan la totalidad de las experiencias existentes en América Latina y el Caribe. Ello no implica que no existan registros institucionales, como los existentes en algunos hospitales de Chile (región del Maule), Ecuador y Perú, que forman parte del ECLAMC e informan a ese programa de investigación (15). Dado que por el momento son pocos los sistemas nacionales que publican o comparten sus datos, no fue posible comparar la información de los sistemas analizados con lo informado en la literatura.

Debido a que los responsables de responder la encuesta fueron los referentes nacionales de salud perinatal, que podían ofrecer una valoración real de la implementación de los sistemas de vigilancia analizados, no debe haber subregistro o distorsión de la información.

El presente estudio constituye la primera exploración exhaustiva de los sistemas nacionales de vigilancia de anomalías congénitas en América Latina y el Caribe y ofrece información relevante y reconocida por las autoridades sanitarias de los países participantes. Sus resultados permiten evaluar los avances e identificar los desafíos relacionados con la expansión de los sistemas nacionales de vigilancia y su armonización. Estos datos regionales, consolidados y sistematizados, permiten también valorar las tendencias y preparar respuestas informadas y oportunas ante situaciones emergentes.

En conclusión, se comprobó la aún escasa disponibilidad y elevada heterogeneidad de los sistemas nacionales de vigilancia de anomalías congénitas en América Latina y el Caribe. Es prioritario avanzar hacia la expansión y el fortalecimiento de la vigilancia de las anomalías congénitas en nuestros países.

Se recomienda trabajar de manera específica, según las características de cada caso, para implementar esos sistemas en los países que aún no cuentan con ellos y fortalecerlos en los países que ya tienen sus registros. Se debe trabajar para lograr la total armonización de los sistemas nacionales y establecer los mecanismos para contar con datos que contribuyan no solo a la vigilancia y la toma de decisiones internas en los países, sino también desde una perspectiva regional.

## Contribución de los autores.

Los autores PD, SS, LADF y AGT concibieron el estudio original; PD, RL, PB, MPB, BG participaron directamente en la recolección y análisis de los datos; todos los autores participaron en la planificación, la interpretación, la discusión de los resultados y la redacción del manuscrito. Todos los autores revisaron y aprobaron la versión final.

## Declaración.

Las opiniones expresadas en este manuscrito son responsabilidad de los autores y no reflejan necesariamente los criterios ni la política de la RPSP/ PAJPH y/o de la OPS.
